# The global burden and epidemiology of invasive non-typhoidal *Salmonella* infections

**DOI:** 10.1080/21645515.2018.1504717

**Published:** 2018-09-05

**Authors:** Ruchita Balasubramanian, Justin Im, Jung-Seok Lee, Hyon Jin Jeon, Ondari D. Mogeni, Jerome H. Kim, Raphaël Rakotozandrindrainy, Stephen Baker, Florian Marks

**Affiliations:** a Princeton University, Princeton, NJ, USA; b International Vaccine Institute, Seoul, Republic of Korea; c Department of Microbiology, University of Antananarivo, Antananarivo, Madagascar; d The Department of Medicine, University of Cambridge, Cambridge, United Kingdom; e Oxford University Clinical Research Unit, Ho Chi Minh City, Vietnam

**Keywords:** iNTS disease, non-typhoidal Salmonella, Epidemiology, Burden, drug-resistance

## Abstract

Invasive non-typhoidal *Salmonella* (iNTS) disease has emerged as a major public health concern. Yet, understanding of the global burden is incomplete, limited particularly by the breadth of blood culture-based surveillance systems that are able to accurately diagnose the etiology of bacteremia. The accessibility of whole genome sequencing has allowed for genetic characterization of pathogens, shedding light on its evolutionary history and sounding alerts for its future progression. iNTS disease is observed to be a particular threat in sub-Saharan Africa, with a case fatality rate greatly exceeding that of typhoid fever, and commonly affecting infants, young children and immunocompromised adults. While iNTS disease might also be a threat in Asia and Latin America, its burden is not well characterized, primarily owing to the lack of comprehensive reporting in these regions. Drug-resistant *Salmonella enterica (S. enterica)* serovars (e.g. Typhimurium sequence type 313 (ST313)) have emerged as a potential consequence of sustained antibiotic pressure. Genetic analyses have identified distinguished iNTS disease-causing strains that are particularly virulent in certain human host populations. Effective treatment strategies, including vaccination, are necessary; iNTS vaccines targeting the most common *S. enterica* serovars, Typhimurium, Enteritidis and Dublin, are currently in early developmental stages. Funding and political support is needed to promote vaccine development and implementation programs to ultimately reduce the threat of iNTS disease in high risk areas.

## Background

Non-typhoidal *Salmonella* (NTS) serovars have an expansive host range, and human infection by these organisms generally results in self-limiting diarrheal disease.^1^ Estimates suggest that NTS is responsible for 93 million enteric infections and 155,000 associated deaths annually.^1^ Additionally, certain NTS serovars are a major cause of bloodstream infections in specific geographical regions, posing a particular threat to those that are HIV or malaria-infected.^2^ Invasive NTS (iNTS) disease is not typically associated with diarrhea but is accompanied by clinical features comparable to other non-specific febrile illnesses, such as typhoid fever, and includes symptoms of fever, hepatosplenomegaly and respiratory symptoms.^3–4^ If left untreated, iNTS disease is often fatal.^3–4^ Immunocompromised individuals, including those infected with HIV and malaria, and infants and young adults living in areas where malnutrition is common, are particularly at risk of acquiring iNTS disease.^1–5^ The *Salmonella enterica (S. enterica)* serovars most commonly associated with iNTS are *S*. Typhimurium, *S*. Enteritidis, and *S*. Dublin.^3,4^ The rising magnitude of iNTS disease has only become acutely apparent in recent decades, which may reflect a change in epidemiology or the parallel emergence of more precise reporting during this period.^5^ A summary of recent studies reporting this disease can provide a better picture of the spread and magnitude of the burden of iNTS disease.

## Epidemiology

Invasive NTS (iNTS) disease has been highly reported in parts of sub-Saharan Africa.^3,4,6,7^ However, there is a general paucity of data regarding the incidence of iNTS disease in many other regions. The lack of data stems largely from the difficulty to accurately diagnose the disease, as this requires laboratories capable of microbiology and trained technicians, both of which are challenging to maintain in resource-limited settings. Ao et al. estimated the global burden of iNTS disease to be 3.4 million cases and 681,316 deaths annually, with almost 2/3 (63.7%) of all cases occurring in children under the age of five.^5^ This study also estimated the incidence of iNTS disease at a regional level. Sub-Saharan Africa and Europe were calculated to have the highest incidences of iNTS disease, with 227 and 102 cases of iNTS per 100,000 population, respectively. Further investigation of the unpredictably high incidence originating from Europe revealed that this was primarily driven by cases from Russia, the Ukraine, and Estonia, which had considerably higher incidences than Western European countries. Notably, the Americas and Southeast Asia had considerably lower incidences of iNTS disease (23 and 21 cases/100,000 population, respectively) and the Middle East and North Africa were jointly found to have almost negligible incidence of 0.8 cases/100,000 population.^5^

**Figure 1.**
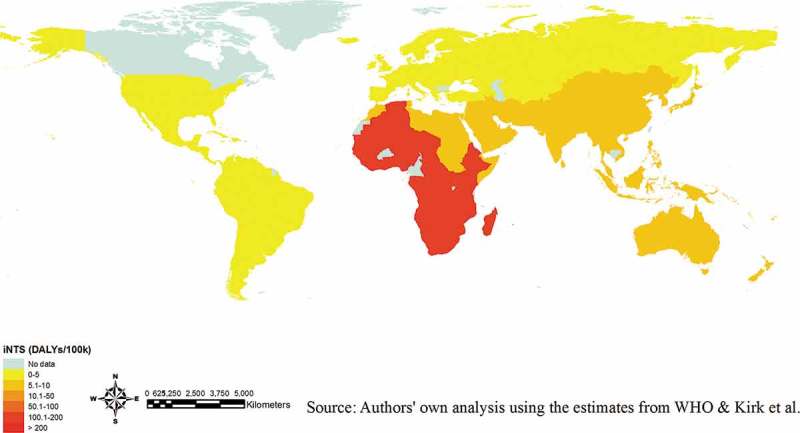
Disability-adjusted life years (DALYs) due to iNTS disease (2010). **NOTE**. Estimates only available at WHO regional-level; map should not be interpreted at the country-level. The full list of the WHO regional categorization (available in ref. 6) The DALY estimates may be conservative due to the exclusion of HIV infected individuals in the analysis.^7^

The World Health Organization (WHO) along with Kirk et al. further assessed the geographical distribution of iNTS disease (Figure 1).^6–7^ These data do not stem from country-level assessments, but rather from WHO regional-level categorization. Kirk et al. constructed a random effect log linear model to estimate the global iNTS incidence.^7^ Due to the scarcity of data, incidences were predicted for all ages, and age-specific incidence rates were derived based on age profiles for iNTS disease cases in low (i.e. United States) and high (i.e. Mali) burden settings.^5^ According to this approach, iNTS disease is more prevalent in Africa in comparison to other parts of the world. However, while these modelled data are helpful in understanding the geographical variation of the disease, the sources of data were sparse, resulting in the use of predicted incidences within some age groups.

More accurate burden estimates of iNTS disease, especially in young children and infants, have only recently become available through population-based surveillance aided by whole genome sequencing to better characterize NTS pathogens.^3^ Further studies are aimed at elucidating the underlying causes of the apparent geographic distribution of iNTS, where potential associations include environmental risk factors and gaps in surveillance infrastructure that mask the presence of iNTS in areas with limited diagnostics or surveillance.

A review focusing specifically on Africa comprehensively reported the burden of iNTS disease by building on the efforts of Ao et al, which restricted their analysis to 14 incidence reports alone, to obtain a more extensive picture of the epidemiology of iNTS disease in Africa from 1966 to 2014.^4^ A total of 176 studies explicitly reporting NTS isolated by blood culture were included, where studies ranged from longitudinal, community-based surveillance to reports of isolates obtained from patients in a hospital or community setting. Information regarding incidence were extracted directly and summarized according to their specific sub-group classification, the study location, the subjects included in the study and the time frame of the study. Published case fatality rates (CFR) were extracted when available and the proportion of bacteremia caused by NTS was calculated. They observed that iNTS disease was focal, with the prevalence of community-acquired bacteraemia associated with iNTS disease ranging from 8% in South Africa and Nigeria to 45% in the Central African Republic.^4^ They additionally found that the earliest reported cases of iNTS disease in Africa dated back to the mid-1960s but 55.7% of all available reports were published between 2005 and 2014, with a peak of 18 reports in 2011. By 2014, iNTS disease had been reported in 33 countries across five geographical regions in Africa, with 53% of the reports originating from Eastern Africa.^4^ iNTS disease incidences were extracted from 14 studies and varied between 1.4/100,000 person-years of observation (PYO) in South Africa, to 855/100,000 PYO in southwest Uganda for people of all ages, where the estimated incidences were the highest among those infected with HIV, those with sickle cell disease, and children.^4^ The highest reported incidence of 2,520/100,000 PYO in children under 5 years of age was localized to rural Ghana.^4^ Extractable CFR data was available from 24 of the included studies, leading to an estimate of an average case fatality rate of 20.6%. This estimate is in accordance with previous observations and was considerably higher than that of the global estimate of CFR for typhoid fever (1%), most likely owing to the fact that the iNTS disease burden is concentrated in vulnerable populations, including young children with co-morbidities.^4^ In sub-Saharan Africa, iNTS disease exhibits a CFR between 20 to 25% and the prevalence of disease is associated with the concurrent endemicity of malaria, a potential risk factor for infection.^8^ Ultimately, these statistics serve the greater purpose of outlining the nature of the threat of iNTS disease in Africa, particularly in sub-Saharan Africa. However, despite the comprehensive nature of these studies, there was a dearth of information from specific regions of Africa, including West Africa. Because countries neighboring West African nations reported cases of iNTS, the lack of published data in West Africa is likely not due to a lack of disease burden but rather the lack of reporting.

To address these gaps in epidemiological data on iNTS disease and the lack of standardized population-based incidence studies in sub-Saharan Africa, the Typhoid Fever Surveillance in Africa Program (TSAP), a multi-country investigation of the burden of invasive bacterial pathogens (particularly typhoid fever and iNTS disease) was conducted from 2010 to 2014.^9–10^ Population-based surveillance yielded disease incidence estimates from 13 sites in 10 sub-Saharan African countries.^10^ Out of all isolated bacteria from blood from patients recruited in all countries, 17% (94/568) were NTS isolates. The overall adjusted incidence rate (AIR) of iNTS disease per 100,000 PYO varied between 0 in Ethiopia, Sudan, Madagascar, and South Africa to 237 in Burkina Faso. Notably, the incidence was generally higher in febrile patients aged less than 5 years^11^. Similarly, AIR rates were specifically higher among infants and young children, with incidences varying between 291/100,000 PYO in Guinea-Bissau to 1,733/100,000 PYO in Ghana in children <2 years of age, confirming previous studies suggesting a higher burden of iNTS disease in young children and contradicting the preconceived notion that iNTS disease shared a similar risk profile with other invasive bacterial infections.^10,11^ Particularly noteworthy was the observation of an association between iNTS disease and *P. falciparum* malaria, where higher iNTS disease incidence was found in areas where *P. falciparum* malaria was endemic.^2^ The association with malaria was further strengthened by a case-control study conducted in two Ghanaian hospitals.^12^ Additionally, in Ghana, a higher incidence of disease was also identified in children from rural locations, as opposed to urban settings.^13^ Data from the TSAP study, in conjunction with other reports, underscore the previously overlooked burden of iNTS disease in the African region and the associated risk factors.

The threat of iNTS disease extends to other WHO regions beyond Africa, albeit to a lesser extent. Previously, little investigation into the burden of iNTS disease in Asia outside of the context of typhoid fever had been conducted. One large-scale population-based fever surveillance study conducted in five Asian countries reported only six iNTS disease cases out of 20,537 enrolled patients with fever.^14^ By site, the incidence of iNTS disease ranged between 1.0/100,000 PYO in North Jakarta, Indonesia and 1.8/100,000 PYO in Kolkata, India, across all age groups.^14^ The highest incidence reported was found in Karachi, Pakistan, with an incidence of 7.2/100,000 PYO among 2–15 years olds.^14^ Recent smaller scale studies have additionally investigated the burden of iNTS disease in Southeast Asian countries. A study characterizing clinical samples collected from human populations, specifically workers with occupations in the meat-handling industry, found 34.6% and 47.4% of samples collected to be positive for *Salmonella i*n Thailand and the Lao People’s Democratic Republic, respectively.^15^ In addition to this high prevalence of *Salmonella*, further molecular characterization of these *Salmonella* isolates revealed *S*. Typhimurium to be the most common serotype (34% of isolates in Thailand and 20.6% of isolates in the Lao PDR), hinting at the potential threat of iNTS disease in these regions. A more recent study conducted in the Lao People’s Democratic Republic utilized multi-locus sequencing typing to characterize the NTS organisms isolated from blood and fecal samples between 2002 and 2012.^16^ Here, the authors found that *S*. Enteritidis, *S*. Typhimurium and *S*. Choleraesuis accounted for >90% (58/63) of iNTS disease cases over this period.^15^ Notably, *S. enterica* Typhimurium sequence type (ST)19 and ST34 were isolated, corroborating previous evidence that described these ST’s in relation to invasive disease in Southeast Asia. A study on sepsis conducted in Thailand, Indonesia and Vietnam found 20/750 (2.7%) of sepsis patients to be positive for iNTS.^17^ While the prevalence of iNTS disease-causing organisms was nontrivial, as all organisms included in this study were collected from patients presenting to health care facilities during routine testing or in the course of a study, the data collected was likely not representative of the nation as a whole and the incidence of iNTS disease could not be determined. However, as the isolation rate of iNTS disease-causing organisms was fairly low, it is likely that the burden of iNTS disease, while not insignificant, does not rival that of sub-Saharan Africa. These data were augmented by a study in Vietnam, where 102 cases of iNTS disease infections were documented between 2008 and 2013, the majority (71%) of whom were also HIV infected.^18^ Cases of iNTS were retrospectively identified through confirmation from blood culture from an infectious disease hospital in southern Vietnam that acts as the main primary and secondary health care facility for the surrounding local population. While iNTS infection was not as common as compared to African settings, a mortality rate of 26% was determined, which is comparable to the estimated CFR in Africa, where HIV infection has been shown to be associated with iNTS disease-related death.^18^ However, as children with HIV were generally referred to a pediatric hospital that was distinct from the study site, the burden of iNTS disease was likely underestimated among the child populations. Regardless, this study supplied the largest description of iNTS disease cases in Southeast Asia to date, highlighting similarities in iNTS disease epidemiology between Asia and Africa, where in the latter setting, iNTS disease has been more thoroughly described. Similarly, in Bangladesh, a CFR of 25% was observed for NTS bacteraemia patients hospitalized between 2009 and 2013.^19^ In Malaysia, a retrospective study conducted from 2001 to 2011 analyzed the clinical details of children aged 13 years or younger with positive blood culture admitted to the neonatal or pediatric wards of a government hospital situated in Selangor, Malaysia.^20^ Of the aetiological agents isolated from patients that were confirmed to have community-acquired bacteremia, one of the most common isolates found was NTS (16.2%), highlighting these organisms’ potential to be a major public health concern.^20^ Collectively, these reports are suggestive of the endemicity of iNTS disease in parts of South and Southeast Asia where iNTS has previously been overlooked, and encourage future efforts to establish surveillance to monitor the presence of iNTS disease-causing organisms and the necessity for vaccines as a preventative measure.

In Latin America, however, there is a severe lack of understanding of the burden of iNTS due to the absence of a systematic large-scale surveillance in this region. In Colombia, a surveillance study conducted over a 6-year period investigated *S. enterica* isolates collected from blood and faecal samples and identified 32.5% of these isolates to be *S*. Typhimurium.^21^ Of the 1,302 *S*. Tyhpimurium isolates identified, 19.5% were obtained from blood culture. While this study did provide insight into the distribution of iNTS disease-causing isolates in Columbia, there were few other studies conducted in this region, and therefore, further standardized investigation of iNTS disease is warranted to obtain a better estimate of the burden in this area.

## Multi-drug resistance/iNTS disease phylogeny

As previously described, the *Salmonella* serovars Enteritidis, Dublin and Typhimurium are most commonly associated with iNTS disease. *S*. Typhimurium is known to infect multiple hosts while *S*. Dublin is more host-adapted to humans.^22^ Recent investigation has suggested that multi drug-resistant (MDR) *S. enterica* serovars, potentially arising due to selective pressure from sustained antibiotic exposure, are more likely to be the causative agents of iNTS disease.^22^ In rural Ghana, *S. enterica* serovars associated with human infection were found to be genetically distinct from those isolated from water well samples in the same setting.^23^ This observation suggests that the *S. enterica* variants associated with iNTS disease are likely able to spread between humans through the faecal-oral route in addition to transmission through a zoonotic reservoir. Of particular concern is the emergence of *S*. Typhimurium sequence type 313 (ST313), which has evolved to cause more invasive disease in humans compared to other sequence types. This clone is potentially more likely to be faecal-orally transmitted and has acquired antimicrobial resistance (AMR) genes producing a MDR phenotype.^24–26^ While ST313 is prevalent in Africa, a clone exhibiting a differing MDR profile than its African counterpart has recently emerged in Brazil.^27^ Whole genome sequencing revealed that two lineages of ST313 are associated with human disease and are adapted to be specifically pathogenic in immuno-compromised humans.^22^ Additionally, *S*. Typhimurium sequence type 19 (ST19) is also an emergent clone that is associated with human disease and is chiefly found in Europe and North America.^8^ *In vitro* experimentation, comparing the pathogenicity of ST313 and ST19, has shown that ST313 is more efficiently phagocytosed and more resistant to killing by human macrophages.^25^ Further investigation of the ST313 genome sequence revealed that this ST contains various pseudogenes and genetic deletions that show a genetic resemblance to the invasive typhoidal serovars and that a single mutation in a non-coding region accounts for the high virulence of ST313 clones isolated in Africa.^28^ Specifically, in ST313, a single nucleotide polymorphism in the promoter region of the virulence factor gene *pgtE* up-regulated its expression, which could potentially promote bacterial survival during human infection, contributing to its pathogenicity and thereby phenotypically distinguishing this sequence type from ST19. Two lineages of *S*. Enteritidis geographically restricted to Africa contain a virulence plasmid and signatures of genome degradation, indicative of differential host-adaptation^28^. These recent discoveries suggest that these *S. enterica* serovars have adapted within specific human host populations and have evolved to become a more virulent and prominent cause of bloodstream infections.

## iNTS disease vaccines

With the findings that MDR-iNTS organisms are currently responsible for a large proportion of iNTS disease infections in endemic regions, traditional first and second-line antibiotics are failing to combat iNTS disease, warranting the development of a long-lasting prevention strategy, notably vaccine development. Until recently, little investment has been provided for this endeavor, potentially because of the perceived lack of iNTS disease burden in regions like Asia and South America, along with the current recess in malaria incidence. As iNTS disease is known to be associated with *P. falciparum* malaria,^2,12^ the decline in malaria incidence was thought to be accompanied by a concurrent decline in iNTS disease incidence. Additionally, the fact that animals are thought to be the primary reservoir for the transmission of iNTS motivated the primary focus on WASH measures. However, in light of recent evidence of the higher burden of iNTS disease and its significant CFR especially in children, several vaccines, including live-attenuated, subunit-based and recombinant antigen-based vaccines, are currently in development. The majority of these vaccine candidates target *S*. Typhimurium.^29^ However, monovalent and bivalent vaccines targeting *S*. Typhimurium and *S*. Enteritidis are also being developed.^29^ Comprehensive protection would be provided through a Th1-mediated immunity coupled with antibody responses, which can be elicited through live-attenuated vaccines.^30^ However, these vaccines would pose a risk to immunocompromised individuals, who are at high-risk for iNTS disease. For these groups, a killed vaccine would be better suited. However, while killed vaccine candidates are believed to suppress iNTS-bacteremia during the acute phase of infection, they generally do not result in systematic clearance in infected individuals.^30^

Given that the burden of iNTS disease is most prevalent in children aged <5 years, with a sustained burden in the <2 years, vaccine integration within the Expanded Program on Immunization (EPI) would be an efficient strategy, however, further epidemiological evidence to guide optimal scheduling is required. Additonal advancements have also been made with regards to the vaccination technology itself. These include the development of Generalized Modules for Membrane Antigens (GMMA) derived from genetically modified bacteria, which allow for the more efficient delivery of NTS vaccines. This technology results from the shedding of outer membrane proteins from the aforementioned bacteria which contain surface polysaccharides and peripheral membrane proteins in their natural conformation to stimulate the innate immune system.^29^ This delivery system is particularly advantageous because the technology does not require an adjuvant, does not involve complex conjugation, is fairly easy to manufacture and has the potential for high immunogenicity, thus making it particularly advantageous in low income settings. As seen for other vaccines targeting multiple serovars (i.e. pneumococcal vaccines), serotype replacement by non-vaccine-included strains could occur.^31–33^ While there is currently no data supporting this, a trivalent vaccine targeting *S*. Typhimurium*/S*. Dublin/*S*. Enteritidis would provide protection in anticipation of such a potential scenario. Avenues for future advancement include the development of vaccines that simultaneously target both iNTS disease and malaria, a technology motivated by their previously identified association.^2,12^ Such vaccines would be particularly relevant in areas where both iNTS disease and malaria are endemic.^24^ Overall, preliminary advancements on the vaccine development front have been made, but political and financial support is needed to facilitate the continued development of vaccines and implementation of vaccination campaigns to target iNTS disease, particularly in areas of high endemicity.

## Conclusion

iNTS disease has proven to be a major public health concern, especially in sub-Saharan Africa. Here, distinct *S*. Enteritidis clades are circulating that can be associated with iNTS disease.^28^ A potential reason for this might be their ability to be opportunistically invasive in the presence of *P. falciparum* malaria and HIV;^28^ hence, the disease burden is highest in infants and young children potentially resulting in higher CFR. Outside of Africa, other genetic lineages seem to fluctuate that are potentially less invasive^28^ and, moreover, the iNTS disease burden has been less investigated, particularly in remote areas in Latin America and Asia. As such, further investment into surveillance programs in these regions is warranted. These studies would provide further evidence of the associated higher risk of iNTS disease in HIV or malaria-endemic areas. Recent analyses of *S. enterica* serovar genomes have alluded to the possibility that certain *de novo* evolved serovars are adapting to specific human hosts and are no longer present in high quantities in environmental reservoirs. This supports the observation that iNTS disease overall is evolving, likely as a result of selective pressure imposed by sustained antimicrobial use coupled with the presence of immunocompromising co-factors such as HIV and *P. falciparum* malaria. As such, the development of new technologies, such as vaccines, is necessary to help curb the spread of iNTS disease in areas of high risk.

## Disclosure of potential conflicts of interest

No potential conflict of interest was reported by the authors.

## Authors' contributions

RB, JI, FM, SB wrote the first draft of the manuscript. JSL conducted the modeling for the figure and created the map. JI, HJJ, ODM, JSL, JHK, RR critically revised the manuscript. All authors agree to its final content.
